# Yield and Composition of the Essential Oil of the *Opopanax* Genus in Turkey

**DOI:** 10.3390/molecules28073055

**Published:** 2023-03-29

**Authors:** Ebru Yüce Babacan, Azize Demirpolat, Uğur Çakılcıoğlu, Eyüp Bagcı

**Affiliations:** 1 Department of Botany, Pertek Sakine Genç Vocational School, Munzur University, 62000 Tunceli, Turkey; 2 Vocational School of Food Agriculture and Livestock, Bingöl University, 12000 Bingöl, Turkey; 3 Department of Biology, Faculty of Sciences, Fırat University, 23119 Elazığ, Turkey

**Keywords:** *Opopanax* genus, essential oil, chemical composition, GC-MS

## Abstract

The genus *Opopanax* W. Koch (Apiaceae) is represented by four species in Turkey. The composition of the essential oil of *Opopanax* genus members (Apiaceae) growing in Turkey was investigated in this study. GC-MS was used to analyze the composition of *Opopanax* essential oil samples that were taken from their natural environments. The Clevenger apparatus was used to hydrodistill the plant’s aerial parts, and the yields were determined to be between 0.2% *v*/*w* (for *O. siifolius*) and 0.4% (for *O. hispidus, O. chironium*, and *O. persicus)*. The results and the chemical data provided some information and clues on the chemotaxonomy of the genus *Opopanax*. In this study, γ-elemene, butanoic acid octyl ester, and cylopropane were the main compounds identified in the essential oils of *O. chironium*, *O. hispidus*, and *O. persicus*. In particular, hexynyl n-valerate was most abundant in the essential oil of *O. chironium*, cyclopropane in that of *O. hispidus*, *γ*-elemene in that of *O. persicus*, and n-hexadecanoic acid/palmitic acid in that of *O. siifolius*. In a chemotaxonomic approach, the essential oil analysis of the *Opopanax* species revealed that these species conformed in a cluster analysis with their morphological classification. The constituents of the essential oils of all examined in the genus *Opopanax* were determined in this study, which is the most thorough one to date. This study provides new information about the composition of the essential oils of the investigated species.

## 1. Introduction

Apiaceae family members are significant plants that have economic importance all over the world. They are used as a vegetable food and as animal feed. Some Apiaceae species are also used as ornamental plants in parks and gardens. Because of the alkaloids and resins they contain, these plants are widely used in the medicine and cosmetics industries as well [1].

*Opopanax* is a genus within the Apiaceae family that is represented in Flora Iranica by *O. persicus* Boiss & Held and *O. hispidus* Griseb. The only species in Siberian flora is *O. armeniacum* Bordz., whereas Flora Europaea includes *O. hispidus* and *O. chironium* W.D.J. Koch [2,3]. The three species identified in Turkey are members of the genus *Opopanax*, which is spread in the Mediterranean region, Western Asia, and southern Europe. The uncommon monotypic Turkish genus *Crenosciadium* was recently classified as a synonym for *Opopanax*. The genus *Opopanax* is also represented by three species in Turkey [4]. As a result of recent studies, *Crenosciadium* Boiss. & Heldr. is a genus that has been combined with *Opopanax* and is represented by four species [5,6]. Bentham & Hooker [7] showed that *Crenosciadium* is closely related to *Opopanax*, while Menemen reported that *C. siifolium* (Boiss et. Heldr) [5] Menemen is a synonym for *Opopanax siifolius* (Boiss. & Heldr.); however, it differs from *Opopanax* in that it has a dorsal flat fruit. Özcan et al. studied the molecular characterizations of the nrDNA ITS, cpDNA matK, and trnL-F regions of *C. siifolium* and *Opopanax* species and determined that these two genera, although very similar, are different from each other [8]. The mericarp morphology is particularly helpful for separating the genera *Opopanax* and *Crenosciadium*, and the mericarp ribs are very significant for separating the two genera [9]. 

Members of the *Opopanax* genus have been used as medicine since antiquity and have also been consumed as a perfume for a long period in the past, for thousands of years. Humans may consume it in a variety of ways, such as by combining it with warm water or consuming it as resin [10]. *Opopanax hispidus* has a wider geographical distribution than other *Opopanax* members. This species is distributed from Western Europe to the Balkans and western parts of Asia. The stem and leaves of *O. hispidus* are used as antiseptics in Iranian ethnobotany, while the stem is used to treat infertility in Turkish folk medicine [11]. The root and aerial parts of *O. chironium* were extensively studied and found to contain various phthalides and C-17 acetylenes. In addition, it has been reported that this species has high antimicrobial and antioxidant activity against *Escherichia coli* and *Listeria monocytogenes* because of the phenols and flavonoids it contains [12]. Previous bioactivity and phytochemical studies showed that the genus *Opopanax* generally produces phenolics, diterpenes, coumarins, phthalides and has diverse biological properties. These compounds provide it with some pharmacological properties such as antioxidant, antimicrobial, and anticancer activities [12,13].

Studies have been conducted on the phytochemistry, conventional use, and pharmacological properties of the *Opopanax* genus. Additionally, data and chemical indicators that the species of this genus could be used therapeutically have been published. The genus *Opopanax* primarily produces phenolics, coumarins, phthalides, and diterpenes and possesses a variety of biological and pharmacological characteristics, including antioxidant, anticancer, and antibacterial activities, according to previous phytochemical and bioactivity investigations [14,15].

In phytochemistry and taxonomic studies, applications based on chemotaxonomy, especially predictions to determine the phytochemical content of plants, have gained great importance in recent years. Investigating the phytochemical components of *Opopanax* species naturally grown in Turkey was the goal of this study. We also aimed to determine the chemotaxonomic relationships between inter and infrageneric means. The results provide important clues on the taxonomic position of the genus members. 

## 2. Results

There were quantitative and qualitative variations in the essential oils of the *Opopanax* species. In the essential oil composition of the *Opopanax* genus naturally grown in Turkey forty-one, thirty-two, twenty-three, and thirty components were identified in the essential oils of *O. chironium*, *O. hispidus*, *O. siifolius*, and *O. persicus*, respectively (Table 1). The identified compounds accounted for 86.9%, 90.8%, 96.4%, and 88.3% of the essential oils of *O. chironium*, *O. hispidus*, *O. siifolius*, and *O. persicus* species, respectively. The essential oil yields varied between 0.2 and 0.4 mL per 100 g of plant sample. 

Hexyl n-valerate was also found to be highly present in *O. chironium* essential oil (18.5%). The presence of myristicin (16.5%), *γ*-elemene (16.0%), and butanoic acide-octyl ester (12.0%) was also important. In the oil of *O. hispidus*, cylopropane was the major compound (24.0%), followed by *γ*-elemene (14.0%), butanoic acid-octyl ester (11.5%), 1,3-benzodioxole (10.5%), and caryophyllene oxide (5.4%).

The results of the essential oils from the aerial part of *O. siifolius* analysis indicated n-hexadecanoic acid/palmitic acid (33.3%), stearic acid/n-octadecanoic acid (17.2%), oleic acid/(*Z*)-9-octadecenoic acid (12.0%) as the major components. The analysis of the essential oil *O. persicus* indicated that *γ*-elemene (20.5%), cylopropane (17.6%), and butanoic acide-octyl ester (13.5%) were the major components.

## 3. Discussion

The composition of the essential oil of *Opopanax* species in Turkey was compared with those reported in other studies and described in terms of constituents content. The essential oil of hydrodistilled *O. chironium* aerial parts was reported to contain cembrene (14.6%), angelicin (coumarins) (4.5%), *β*-caryophyllene (3.2%), *E-β*-Ionone (3.7%), *(E)-β*-damascenone (3.7%), and *(E)*-geranyl-acetone (3.3%) as main compounds (59). The only diterpenes detected in the extracts of *O. chironium* and *O. persicum* were coumarins. These species are also endemic to Iran, Turkey, Iraq, and Transcaucasia [57,58,59]. The presence of coumarins, such as columbianadin, eucedanin, diterpene, and gaudichaudin, called peucelinenoxide acetate, was determined in the extract obtained from *O. chironium* [13,60,61]. In the oil of *O. chironium* (L.) Koch, *E*-farnesyl acetate (20.2%) was determined as a major component [62]. In our study, trans-*β*-farnesene was found at a minor level (0.3%).

In the essential oil from the fruits of *O. hispidus*, 79 compounds were identified, and geranyl acetate (17.9%), tyrosol (48.8%), incensole acetate (4.3%), and germacrene D (4.0%) were the main components [63]. The content of geranyl acetate was 1.0% and that of germacrene D was 0.5% on the essential oil, and they appeared as minor components in this study. The content of *γ*-elemene was determined to be 20.5% in our study, and thus, it was found to be a significant chemical. *γ*-elemene from *O. hispidus* has shown strong insecticidal activity against the agricultural pest *Spodoptera litura* and may be beneficial as an environmentally benign biopesticide [64]. *O. hispidus*, used for the treatment of sclerosis, can also be used as a biopesticide [65]. The other major component of the essential oil from the *Opopanax* genus determined in this study was cyclopropane, found in the essential oils of *O. chironium*, *O. hispidus*, *O. persicus* in quantities corresponding to 5.5, 24.0, and 17.6%, respectively. 

The oils of *O. hispidus*, *O. chironium*, and *O. persicus* were found to contain butanoic acide-octyl ester (content of 11.5%, 12.0%, and 13.5%, respectively. A fatty acid found naturally in plants is butanoic acid octyl ester. It can be found as a fatty acid and a glycol ether. *Candida glabrata*, a fungus that can infect the skin, has been demonstrated to be resistant to butanoic acid octyl ester. Butanoic acid octyl ester has been used as a dietary supplement to increase the absorption of lipophilic nutrients, such as vitamins A, D, E, and K. There are no documented side effects or toxicity for this substance [66].

In this study, the lowest amount of n hexadecanoic acid (33.3%) was found in the essential oil of *O. siifolius*. Due to the harmful side effects of synthetic compounds used for chemical protection, the use of substances obtained from plant sources, showing antimicrobial activity, is increasing. Palmitic acid, also known as n-hexadecanoic acid, is a type of saturated fatty acid [67]. It is well known that several fatty acids have antibacterial and antifungal effects [68]. By directly interacting with T cells, fatty acids can influence the immunological responses [69]. By reducing the generation of inflammatory mediators, dietary conjugated linoleic acid has an anti-inflammatory impact [70].

The essential oil analysis performed here showed that all *Opopanax* species are rich in sesquiterpenes and low in monoterpenes. Especially, *O. siifolius* contains more common saturated and unsaturated fatty acids. The results demonstrated the occurrence of major compounds in the genus patterns, i.e., the hexynyl n-valerate chemotype in *O. chironium*, the cyclopropane chemotype in *O. hispidus*, the *γ*-element chemotype in *O. persicus*, the palmitic acid chemotype in *O. siifolius*, in the eastern Anatolian region of Turkey. The chemical analysis results of *O. siifolius* showed that this species may be a different group and could be transferred to a genus different from *Crenosciadium.* As in our previous studies, the chemical analysis results supported the morphological data [71,72].

**Figure 1:**
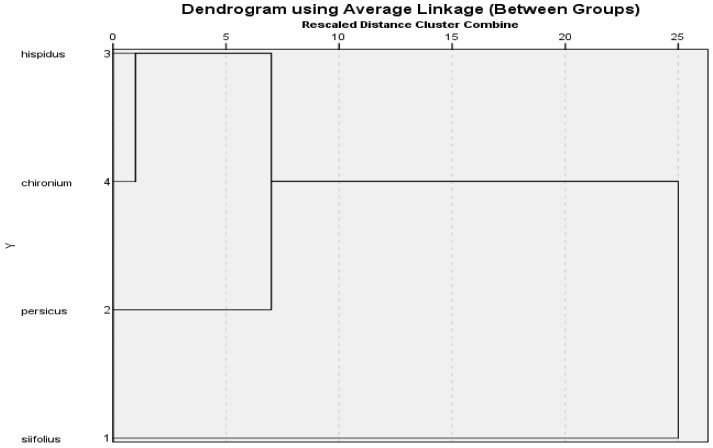
Clustering analysis of the *Opopanax* genus according to oil volatile components.

As shown in Table 1, the essential oil analysis conducted for this study indicated some chemical differences and similarities among the members of the *Opopanax* genus. The result of the clustering analysis was based on 10 main components in the essential oils of the *Opopanax* spp. examined, (Figure 1) and revealed that the *Opopanax* species were chemically as well as morphologically similar. The cluster analysis showed that *O. siifolius* belongs to the outermost clade according to the main essential oil compounds. Considering the groupings, it was observed that *O. chironium*, *O. hispidus*, *O. persicus* species were connected to each other (big group), and it is said that, chemically, they are closely related species. However, *O. siifolius* was far from these three species and appeared as a single species. It was concluded that the results are compatible with the morphological appearance of the taxa. The dendrogram showed that *O. chironium* and *O. hispidus* are closely related species, and *O. persicus* is connected with this small cluster and has a high similarity. *O. siifolius* was found in a different single cluster that combines with this triple cluster at a high level. This chemical grouping of the *Opopanax* genus members confirmed that *O. siifolium* may belong to a different genus, as indicated by the morphological analysis [5] and the molecular characterization of the *Opopanax* and *Crenasciadum* genera. 

The results and the chemical data provided some information and clues on the chemotaxonomy of the genus *Opopanax*. In this study, *γ*-elemene, butanoic acid-octyl ester, cylopropane were the main components determined in *O. chironium*, *O. hispidus*, *O. persicus* whereas palmitic acid was the dominant component in *O. siifolius*. Hexynyl n-valerate (*O. chironium*), cyclopropane (*O. hispidus*), *γ*-elemene (*O. persicus*), and palmitic acid (*O. siifolius*) were the most abundant components in the essential oils of the analyzed taxa. Myristicin was detected only in *O. chironium*, and spatulenol was detected only in *O. hispidus*. Hexyl n-valerate, *γ*-elemene, butanoic acid-octyl ester and cyclopropane were among the major components in *O. chironium*, *O. hispidus*, and *O. persicus* oils. On the other hand, the major compounds detected in *O. siifolius* were different from those identified in the other *Opopanax* species studied (palmitic, stearic, oleic, linoleic acids and ethillinoleolate) (Table 1).

**Figure 2:**
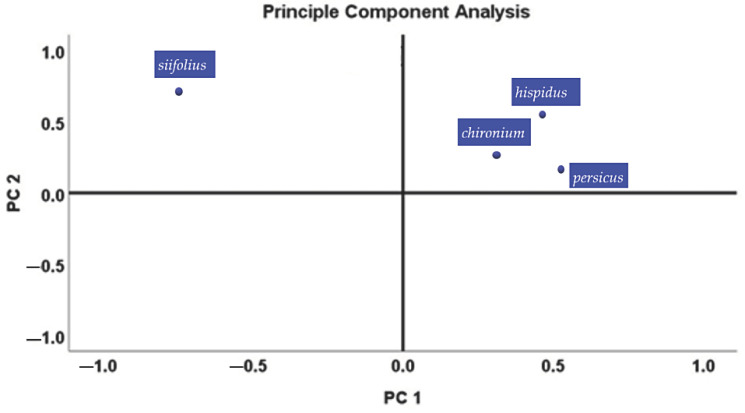
The composition of the essential oils of *Opoponax* species was analyzed using principal component analysis (PCA).

**Figure 3:**
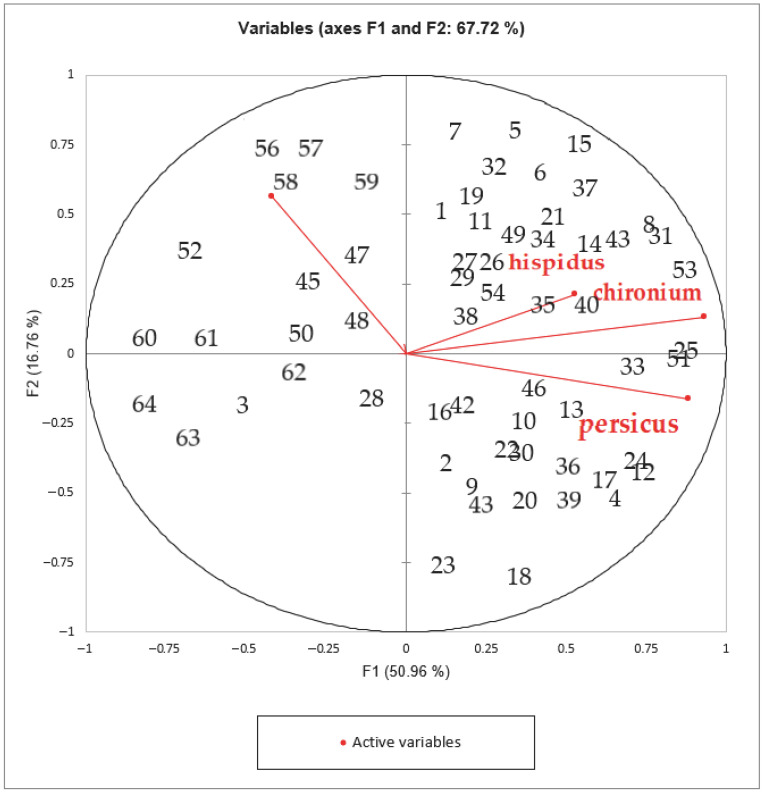
Biplot (PCA) of the composition of the essential oils of the examined *Opopanax* species.

Based on previously published investigations, multivariate analysis was applied [29]. Both principal component analysis (PCA) and cluster analysis (CA) were used to determine the chemicals in the various samples. Then, using the matrix correlation setup, PCA was carried out using Varimax rotation. PC1 (50.96%) and PC2 (16.76%) were the two primary components of the principal component analysis, respectively. PC1 and PC2 carried a combined load of 67.72%. To investigate the correlation between the variables, the Kaiser–Meyer–Olkin (KMO) approach was used. The KMO value was 0.728, which is a respectable value. For the data set, Barlett’s test of sphericity likewise demonstrated a statistically significant difference at alpha of 0.04. The link between the four *Opopanax* species and their essential oil concentration was highlighted using PCA analysis, which was carried out using two different groups (PC1 and PC2). Figure 1, Figure 2 and Figure 3 show the results.

A biplot graph was created in this work to establish the multivariate relationships of the chemicals in the essential oils of the four *Opopanax* species investigated (Figure 3). If the angle between the vectors was less than 90°, the content of that species was better than the average; if the angle was larger than 90°, the content of that species was lower than the average; if the angle was equal to 90°, the content was near the average. Figure 3 shows the values of all components in the examined species and if they properties were positively or negatively associated with one another. The statistical analyses verified the results.

Essential oils have a wide range of beneficial properties. In this work, we isolated the essential oil from species of the *Opopanax* genus. It is possible to say that it is useful to compare the composition of essential oils and to provide basic data for taxonomic and essential oil evaluations in studies on plant genera. The study of essential oils is important to obtain chemotaxonomical relationships and promote scientific agriculture and product diversity. It is thought that the information on these essential oils will be useful for many industries working with natural products, such as the medical, cosmetics, landscaping, flavor, and food industries.

## 4. Materials and Methods

### 4.1. Sample Collection

The location information for the plant samples’ natural habitats is provided in Table 2. The plants were taken from their natural environment during the peak flowering season, which is from June to August, and were air-dried in the shade. The aerial parts of the species were used in the analyses. The Fırat University Herbarium is where the plant specimens are kept (FUH).

### 4.2. Isolation of Essential Oils and GC-MS Analysis

The plants used in this research were air-dried. The oil from the plants was extracted using the hydrodistillation technique. Using the Clevenger equipment, 3 h of hydrodistillation were performed on 100 g of air-dried aerial plant material. The organic layer in the gathering vial was transferred to the GC/GC-MS FID equipment once the distillation process was accomplished.

GC-MS was used to examine the essential oils [71,72]. The instrument was an HP 6890 model. The mass range was between 40 and 330 *m*/*z*, and the ionization energy was 70 eV. *Opopanax* samples (1 µL) were loaded into a GC (HP 5890 Series) for the analysis. The samples were run on a multi-purpose HP-5 column, 30 m, 0.25 mm, 0.25 µm (catalog number 19091J-313 Agilent, Santa Clara, CA, USA) [73,74]. 

Helium was used as the carrier gas, with a steady column flow rate of 1 mL/min. The settings for the column oven temperature program were 40 °C and a hold time of 2 min with a temperature rise of 3 °C/min rate until 240 °C (hold time, 2 min.). The flow rate was set to 1 l, and a 3.5 min buffer hold time was applied to the hexane samples. The split mode was chosen (split ratio 1:10 or 1:100). The detector temperature was 250 °C.

The mass spectrometric settings were full scan mode, 20,000 amu/s scan speed, and 50 spectra per sample frequency. The temperature at the contact injector and ion source was 250 °C and 200 °C, respectively.

Alkanes were used as standards to compare the retention indices (RI). By comparing the retention times (RT), mass spectra, and RI of the essential oils to those described in the literature (NIST 20 and Wiley Libraries) and MS libraries (Wiley MS library, New York, NY, USA) [75], the chemical components of the essential oils were identified. Traditional library searches just compare spectra rather than taking the retention parameters into account. In this study, libraries were searched using a combination of storage indexes, which made the compound identification simpler and more accurate. The device’s retention index spectrum libraries were also utilized in this study. The same analytical procedure as that for an identical column provided in the library was applied for better results. Table 1 details the essential oil constituents that were identified.

### 4.3. Cluster Analysis and PCA (Principal Component Analysis)

Ten significant components (less than 1%) were chosen from the water-distilled essential oil components. For the four *Opopanax* species, these elements were submitted to cluster analysis using numerical taxonomic techniques. The UPGMA statistical approach and the IBM SPSS Statistics 21.0.0 software were both employed for this investigation. Dendrograms were used to show the findings of these analyses, and the results were assessed in terms of numerical chemotaxonomic connections. The relationships between the species are shown in the cluster analysis tree shown in Figure 1.

To determine the variability’s organizational structure and calculate the distances between groups, multivariate analysis was used. Complete data sets were used for these analyses. To determine the commonalities between each measured unit, the UPGMA (unweighted pair-group average linkage) clustering approach based on Pearson distances was applied (Figure 2). The chemical components of the four *Opopanax* species’ essential oils served as the dependent variable. The chemicals of various samples were assessed using cluster analysis (CA) and principal component analysis (PCA). The unstandardized statistics were assigned the same weight as that previously reported.

The promax rotation was then used to perform the PCA with the matrix correlation configuration. PCA was then performed in the XLSTAT 2022 software using the matrix type correlation configuration. The species’ proximity and distance from one another based on their essential oils’ content are discussed. “R” (Boston, MA, USA) was used to create biplot graphs (Figure 3).

## Figures and Tables

**Table 1:** Components of the essential oils from species of the *Opopanax* genus.

No.	Components	RI	RI(lit)	Identification Method	% Concentration			
					*O.* *chironium*	*O.* *hispidus*	*O.* *siifolius*	*O.* *persicus*
1.	Octanal	1074	1023 [16]	RI, MS	0.7	-	-	-
2.	Isovaleric acid	1076	872 [17]	RI, MS	0.1	-	-	-
3.	*ο*-Cymene	1090	1026 [18]	RI, MS	0.2	-	0.1	-
4.	*β*-Ocimene	1099	1040 [19]	RI, MS	0.8	0.2	-	0.5
5.	Cyclohexane	1119	1027 [20]	RI, MS	0.3	-	-	-
6.	1-Octanol	1127	1093 [21]	RI, MS	2.0	0.5	-	1.2
7.	Butanoic acid	1146	1140 [22]	RI, MS	0.2	0.2	-	0.4
8.	n-Amyl-Isovalerate	1153	1125 [23]	RI, MS	0.5	0.2	-	0.3
9.	Propanoic acid	1180	1511 [24]	RI, MS	0.1	-	-	1.4
10.	Etenilcyclohexane	1216	825 [25]	RI, MS	0.1	3.0	-	-
11.	Decanal	1220	1204 [26]	RI, MS	-	0.2	-	0.1
12.	Cyclopropane	1224	405 [27]	RI, MS	5.5	**24.0**	-	**17.6**
13.	Hexyl-2-hetylbutanoat	1240	1240 [28]	RI, MS	-	0.5	-	-
14.	Hexyl n-Valerate	1246	1247 [29]	RI, MS	**18.5**	0.5	-	9.5
15.	Pentanoic acid	1249	1720 [30]	RI, MS	1.0	-	-	-
16.	Cis-cyclodecane	1258	1227 [27]	RI, MS	0.2	-	-	-
17.	Cyclobutane	1270	455 [31]	RI, MS	0.2	-	-	-
18.	Acetic acid, decyl ester	1303	1394 [32]	RI, MS	0.1	-	-	-
19.	n-Octyl İso Butyrate	1332	1372 [32]	RI, MS	0.5	0.3	-	0.6
20.	Eugenol	1339	1359 [26]	RI, MS	0.1	-	-	-
21.	Thujene	1358	1033 [33]	RI, MS	1.5	1.5	-	1.7
22.	Bicyclo [4.1.0] heptane	1367	796 [34]	RI, MS	1.3	2.3	-	2.0
23.	Butanoic acid-octyl ester	1370	1372 [35]	RI, MS	**12.0**	**11.5**	-	**13.5**
24.	*α*-Bourbonene	1379	1385 [36]	RI, MS	0.5	-	-	-
25.	1-Decene	1384	1061 [37]	RI, MS	1.5	1.5	-	1.8
26.	Geranyl acetate	1387	1392 [38]	RI, MS	-	1.0	-	0.2
27.	*β*-Caryophyllene	1391	1392 [22]	RI, MS	-	0.8	-	-
28.	Geranyl formate	1404	1400 [22]	RI, MS	-	-	0.5	-
29.	trans-*β*-farnesene	1414	1477 [39]	RI, MS	0.3	0.2	-	0.3
30.	cis-*α*-Bbsabolene	1417	1417 [22]	RI, MS	0.2	-	-	-
31.	Naphthalene	1429	1429 [22]	RI, MS	-	0.3	-	-
32.	Germacrene D	1434	1432 [22]	RI, MS	0.7	0.5	-	0.8
33.	*β*-Gurjunene	1442	1475 [19]	RI, MS	0.2		-	0.5
34.	*γ*-Elemene	1447	1437 [22]	RI, MS	**16.0**	**14.0**	0.9	**20.5**
35.	1,3-Benzodioxole	1458	1531 [27]	RI, MS	0.5	10.5	0.9	5.7
36.	*β*-Sesquiphellandrene	1460	1526 [19]	RI, MS	-	1.1	-	-
37.	Myristicin	1463	1522 [40]	RI, MS	**16.5**	-	-	-
38.	Germacrene B	1483	1482 [22]	RI, MS	1.2	0.7	-	1.2
39.	Spatulenol	1493	1495 [22]	RI, MS	-	3.1	-	-
40.	Caryophyllene oxide	1497	1497 [22]	RI, MS	0.5	1.5	0.4	1.8
41.	Isospathulenol	1525	1638 [41]	RI, MS	0.1	0.5	-	0.3
42.	*α*-Cadinene	1538	1511 [38]	RI, MS	1.5	1.7	-	1.5
43.	Isoaromadendrene epoxide	1548	1594 [41]	RI, MS	-	0.3	1.0	0.1
44.	*σ*-Damascone	1554	1456 [42]	RI, MS	0.3	-	-	-
45.	Farnesol	1625	1742 [43]	RI, MS		-	3.3	-
46.	2 -Pentadecanone	1629	1448 [44]	RI, MS	0.1	2.5	1.5	0.5
47.	1,2-Benzenedicarboxylic acid	1637	1643 [45]	RI, MS	-	-	0.5	-
48.	Pentacosane	1652	1561 [44]	RI, MS	-	-	0.9	
49.	n-Hexadecanoic acid/Palmitic acid	1690	1970 [46]	RI, MS	0.5	2.7	**33.3**	1.8
50.	Adamantane	1712	1400 [47]	RI, MS	-	-	1.2	-
51.	Benzeneacetaldehyde	1741	1640 [48]	RI, MS	0.1	-	-	-
52.	Cembrene A	1747	1955 [49]	RI, MS	-	-	3.8	0.6
53.	Methoxsalen	1749	2020 [50]	RI, MS	0.1			
54.	2-Hexadecen-1-ol	1791	1420 [51]	RI, MS	-	2.1	1.5	
55.	Linoleic acid	1805	2078 [43]	RI, MS	0.1		5.1	0.5
56.	Oleic acid/(Z)-9-Octadecenoic acid	1809	2141 [52]	RI, MS	-	-	**12.0**	-
57.	Etillinoleolat	1812	-	RI, MS	-	-	3.9	-
58.	3-Cyclohexane-1-methanol	1821	1071 [53]	RI, MS	-	-	1.7	-
59.	Stearic acid/n-Octadeca noic acid	1826	2180 [41]	RI, MS	-	-	**17.2**	1.0
60.	Cyclooctanone	1896	-	RI, MS	-	-	2.0	-
61.	Tricosane	1900	1902 [54]	RI, MS	0.1	0.2	-	-
62.	*Cis*-Sesquicyclo-geraniol	1904	1865 [55]	RI, MS	-	-	1.9	-
63.	Trimethylene	1918	-	RI, MS	-	-	1.6	-
64.	Eicosanoic acid	1929	2365 [56]	RI, MS	-	-	0.8	0.1
	**TOTAL**				**86.9**	**90.8**	**96.4**	**88.3**

**Table 2:** Collection date and locality information for the studied *Opopanax* species.

1	*O. chironium*	Tekirdağ between Hayrabolu, 10 km from Tekirdağ, wet places along the river, 70 m., 13 June 2015, *Paksoy* * 2018 N 40.951741, E 27.468926
2	*O. hispidus*	Muğla, Turgutreis Aspat region, Aspat castle and around, within the scrubs, 100–150 m, 2 July 2015, *Paksoy* 2021 N 37.000135, E 27.267143
3	*O. persicus*	Van, Near the Hoşap, wet grassy places, 2000 m, 20 June 2016, *Paksoy* 2049 N 38.317180, E 43.802793
4	*O. siifolius*	Antalya, Akçay, Around the Girdev lake, near the river, 2000 m, 3 August 2015, *Paksoy* 2024 N 36.664530, E 29.659819

* The samples were collected by Mehmet Yavuz Paksoy.

## Data Availability

Not applicable.
